# Carbon-Based Honeycomb Monoliths for Environmental Gas-Phase Applications

**DOI:** 10.3390/ma3021203

**Published:** 2010-02-19

**Authors:** Carlos Moreno-Castilla, Agustín F. Pérez-Cadenas

**Affiliations:** Departamento de Química Inorgánica, Facultad de Ciencias, Universidad de Granada, 18071 Granada, Spain; E-Mail: afperez@ugr.es (A.F.P-C.)

**Keywords:** carbon-coated honeycomb monoliths, integral carbon honeycomb monoliths, NO removal, volatile organic compounds removal

## Abstract

Honeycomb monoliths consist of a large number of parallel channels that provide high contact efficiencies between the monolith and gas flow streams. These structures are used as adsorbents or supports for catalysts when large gas volumes are treated, because they offer very low pressure drop, short diffusion lengths and no obstruction by particulate matter. Carbon-based honeycomb monoliths can be integral or carbon-coated ceramic monoliths, and they take advantage of the versatility of the surface area, pore texture and surface chemistry of carbon materials. Here, we review the preparation methods of these monoliths, their characteristics and environmental applications.

## 1. Introduction

**Figure 1:**
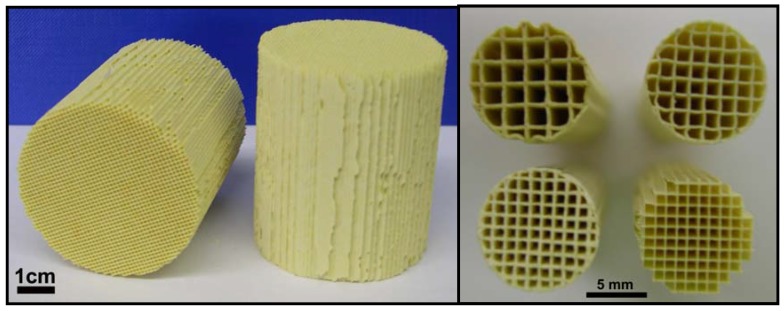
Ceramic honeycomb monoliths with different cell density.

Honeycomb monoliths (HMs) are continuous, unitary ceramic or metallic structures, with long parallel and straight channels extended through the body, which are separated by thin walls, as can be seen in Figure 1. The first monolithic structures had hexagonal-shaped passages that gave a honeycomb appearance to the cross-section of the monolith. Monolithic structures are manufactured today with different channel shapes, although the square shape has reached widespread commercial use since this is the simplest to form.

The main characteristics of HM structures are the high void fraction and the large geometric surface area, which results in a low-pressure drop when high flow rates are used. In addition, they have a high dust tolerance and a large contact area between the monolithic structures and the reactants [1,2,3,4]. All these characteristics make the HMs very useful materials to be applied as adsorbents and support for catalysts in environmental applications. The first important application of ceramic HMs was in the automobile industry as support for catalysts used in the purification of exhaust gases (three-way catalysts). Since then, these ceramic HM materials have found application in the removal of other pollutants [3,5].

The ceramic material most commonly used in the preparation of HMs is synthetic cordierite, a material having a low thermal coefficient of expansion. Its overall chemical composition is about 14 wt % MgO, 36 wt % Al_2_O_3_ and 50 wt % SiO_2_, with small amounts of Na_2_O, Fe_2_O_3_ and CaO. HMs are characterized by their channel or cell shape and size and the wall thickness (Figure 1), which are controlled during the fabrication process. These parameters determine the cell density, that is the number of cells per unit of cross-sectional surface area, and they affect the performance of the monolith. The above parameters can be widely varied, although one of the most commonly used ceramic HMs has about 62 cells/cm^2^, which corresponds to approximately a 1 mm channel width and 0.15 mm wall thickness [1]. HMs generally have homogeneous channels, within the production variance, through the whole body.

The manufacture of ceramic HMs has been described in different reviews in the literature such as references [3,4,5,6]. They are basically made by extrusion of the paste containing cordierite and other processing ingredients, followed by drying and calcination. Ceramic HMs have a ratio of geometric surface area to support volume in the range of 2–4 m^2^/L, which is extremely low to adequately work as an adsorbent or catalyst support. For this reason, these structures have to be coated with a thin layer of other substances that cover the inner surface of the channels and increase the internal surface area. This thin layer is generally called the washcoat. Monoliths prepared in this way are referred to as coated monoliths. The ceramic monoliths gives the mechanical and geometric properties, whereas the washcoat the adsorptive and/or catalytic properties.

Another alternative is to prepare HMs with a unique material that provides all the above properties. These are referred to as integral monoliths. This can be a complicated matter, because a good adsorbent not necessarily gives a monolith with good mechanical properties. So, the preparation of monoliths with new materials needs a great effort to find the adequate conditions for extrusion and immediate conformation in monolithic structures [4].

Carbon materials are known to have good properties as adsorbents for pollutants in the gas and liquid phases [7,8] and also as support for catalysts [9,10]. Therefore, carbon-based HMs are extremely attractive for both applications, particularly if the channel walls are constructed of a carbon material with the surface area, porosity and surface chemistry more appropriate to the required use. In addition, most carbon materials are insensitive to water vapor due to the hydrophobicity of their surface [11,12], which is an advantage for some applications. This paper will review the different methods that have been published to prepare carbon-based honeycomb monoliths, and their current applications as adsorbents and supports for catalysts for the abatement of air pollutants.

## 2. Preparation Methods

Two different types of carbon-based HMs can be distinguished: carbon-coated and integral carbon monoliths. In general, carbon-coated monoliths are mechanically the most resistant; however both types of HMs can be used in different applications. Recently, carbon-coated HMs with very thin coating have shown better performance that the integral ones for liquid-phase processes, since the internal mass diffusion limitations can be reduced [13]. On the contrary, integral carbon HMs could be preferred for gas-phase processes, because high surface areas per total weight (or volume) of monolith can be accessible.

### 2.1. Carbon-coated HMs

Several types of honeycomb monolithic structures prepared from different materials may be coated with well adhered carbon layers. Cordierite based HMs are the most widely used, although other materials as mullite, alumina or clays were investigated [3,4,14,15]. Cordierite HMs are prepared by mixing together various clays with polymeric binders and extruded through steel molds in honeycomb shapes. The honeycombs are then fired to high temperatures (up to 1500 °C) to burn out binders and to react and sinter clays to form cordierite honeycombs. These honeycombs may be fabricated with a wall thickness of 0.1 mm or thicker and with cell densities as high as 95 cells/cm^2^. The most usual cell shape is square, although round channels have been recently obtained from cordierite square-channels by washcoating with α-alumina [16,17]. Adhesion of a carbon layer on the channel surface of a ceramic HM can be carried out by two different methods: dip-coating and chemical vapor deposition (CVD).

#### 2.1.1. Dip-coating

**Figure 2:**
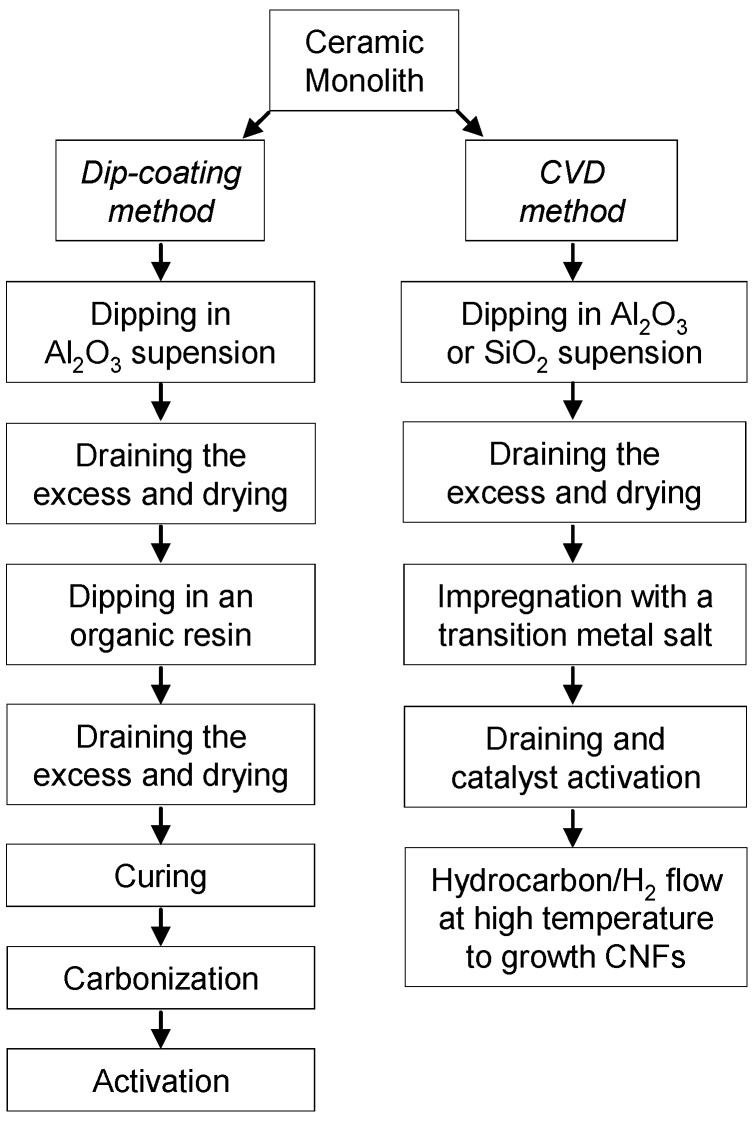
Preparation steps for carbon-coated HMs.

Gadkaree [18] was a pioneer in the preparation of carbon-based HMs by dip-coating a ceramic HM. For this, the ceramic HMs are simply dipped in a resin, allowed to soak for a few minutes and then drained of the resin excess by air blowing through the channels, before being subjected to drying, curing, carbonization and activation processes. The resin used was a phenolic resole due to it had a low viscosity (100 cP) which allows the impregnation and draining step to be carried out easily. In addition, the inexpensive phenolic resins have a very high carbon yield, reducing the cost of the carbon produced. Figure 2 shows a scheme that summarizes the preparation steps of carbon-based HMs following the dip-coating method.

The resins remain on the surface of the channels as a thin layer after the drying and curing steps. Carbonization is carried out at 900 °C in nitrogen flow and the resulting material is CO_2 _activated to 25–30% burn-off. After carbonization, a ceramic-carbon composite structure is formed. This structure is monolithic with carbon forming a continuous coating inseparable from the ceramic backbone. This method can be applied to a large variety of ceramic HMs to obtain carbon-coated HMs with different cell density, wall thickness and cell geometries.

Other authors used the same method developed by Gadkaree to prepare carbon-coated HMs by using different carbon precursors such as phenolic (resole and novolac) and furanic resins, polysaccharides (sucrose and dextrose) and furfuryl alcohol resins [17,19,20,21,22,23].

**Figure 3:**
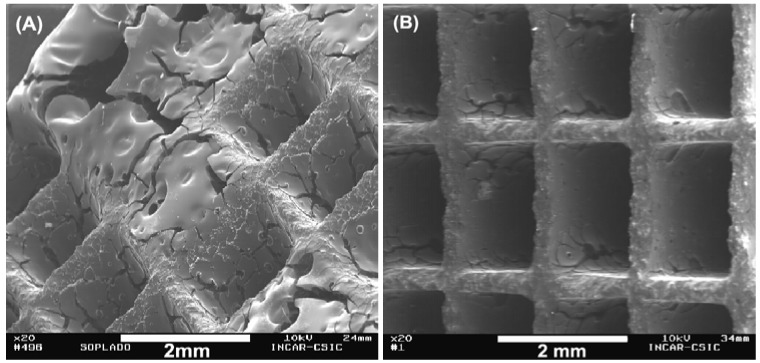
SEM microphotographs of carbon-coated HMs prepared by A: air blowing and B: spinning. From reference [20], with permission from Elsevier.

The effect of the coating and numbers of impregnation cycles was studied by Valdés-Solís *et al*. [20] using a cordierite monolith with 31 cells/cm^2^. A carbon-coated HM with a 14 wt % of C was obtained with a phenolic novolac resin by two impregnation-curing-carbonization cycles. The carbon layer was very uniform due to the fact that the excess of impregnating solution was removed by spinning instead of by air blowing (see Figure 3), and also due to the control of the concentration and viscosity of the resin and the impregnation temperature. Steam activation of the carbon-coated HMs produced materials with BET surface area and micropore volume around 1500 m^2^/g and 0.5 cm^3^/g, respectively. The activated material showed good adsorption properties and electrical conductivity and a high mechanical strength.

Optimization of the dip-coating process of two cordierite HMs using novolac and furan resins was investigated [21,22]. The best viscosity of the dip-coating mixture was found to be 100 cP. Carbonization was completed at 700 ºC for novolac and 600 ºC for furan. Carbon yields and textural properties of resulting carbon-coated HMs depended on the carbon precursor used. An interesting innovation with regard to previous works was that the ceramic HMs were also dip-coated in a slurry of the above resins and a commercial porous activated carbon. Monoliths coated with pure furan resin had less micropore volume and surface area than novolac resin, but the mesoporosity was comparable. The microporosity and especially mesoporosity of the carbon coating increased considerably by adding to these resins a highly porous activated carbon as filler. Coating efficiency was tested by measuring the cordierite resistance to acid leaching. Leaching resistance was greatly improved coating the cordierite HMs with the activated carbon-furan slurry instead of with the furan alone.

When furfuryl alcohol-based polymers were used to dip-coating cordierite HMs (31, 62 and 93 cells/cm^2^) the final carbonization step produced a micro-macroporous carbon coating [23]. Cracks formed during the shrinkage of the polymer upon carbonization gave place to the macropores. Some mesopores were created by a heating step in air at 250 ºC. Shrinkage of the polymer caused incomplete coverage of the cordierite which made the material unsuitable in acidic or alkaline media. Carbonization increased the growing of aromatic structures which increased the electrical conductivity of the carbon layer after carbonization above 700 ºC.

**Figure 4:**
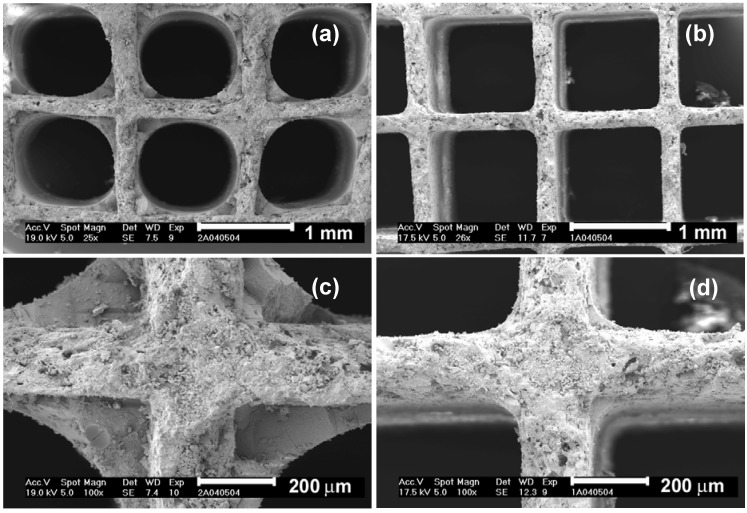
SEM pictures of the channels (cross section) from α-Al_2_O_3_ coated monoliths after the first [(b) and (d)] and the last [(a) and (c)] coating. From reference [17], with permission from Elsevier.

Cordierite HMs have a macroporous wall structure in which an ineffective catalytic active phase can be deposited. To avoid this problem, a square channel cordierite HM with 62 cells/cm^2^ and wall thickness of 0.18 mm was previously dip-coated with α-Al_2_O_3 _before coating with a carbon layer obtained after dip-coating with polyfurfuryl alcohol in a second step [17]. The α-Al_2_O_3 _coated HM had rounded channels as shown in Figure 4, with the cordierite macroporosity blocked. After carbonization of the polymer layer at 350 ºC and air activation at 300 ºC, the carbon obtained had a large oxygen content, some micropores and no mesopores. The presence of an α-Al_2_O_3_ layer between the cordierite wall and the carbon layer prevented the catalyst deposited (Pd) on the carbon layer from penetrating into the walls of the cordierite channels.

A different method to that of the dip-coating seen so far, referred to as melting method [2], was used to prepared carbon-coated HMs [24]. For this, the ceramic HM was mixed with a coal tar pitch and heated under N_2_ flow in a crucible to 800 and 1000 ºC. The excess of carbonized pitch, which remained out of the composite, could be easily removed because it did not adhere to the composite. The composite had a sufficient electrical conductivity for electrochemical applications and stability against oxygen.

#### 2.1.2. CVD

Since Xu and Moulijn [15] described a method based on CVD of hydrocarbons onto an alumina wash-coated HM, several works were published [19,25,26,27,28,29,30] that use transition metals as catalysts to growth carbon nanofibers (CNFs) on HMs from hydrocarbon/H_2_ mixtures. In all cases, the ceramic HMs were previously washcoated with alumina or silica to prevent the CNF growth inside the macropores of the HM destroying the monolith. A scheme that summarizes the preparation steps of carbon-based HMs following the CVD method is depicted in Figure 2. The structure and dimensions of the CNFs were determined by the temperature, gas composition, metal catalyst, metal particle size and nature of the support and washcoat. The support structure is determined by the extent of the fiber entanglement and the individual fiber orientation [25].

**Figure 5:**
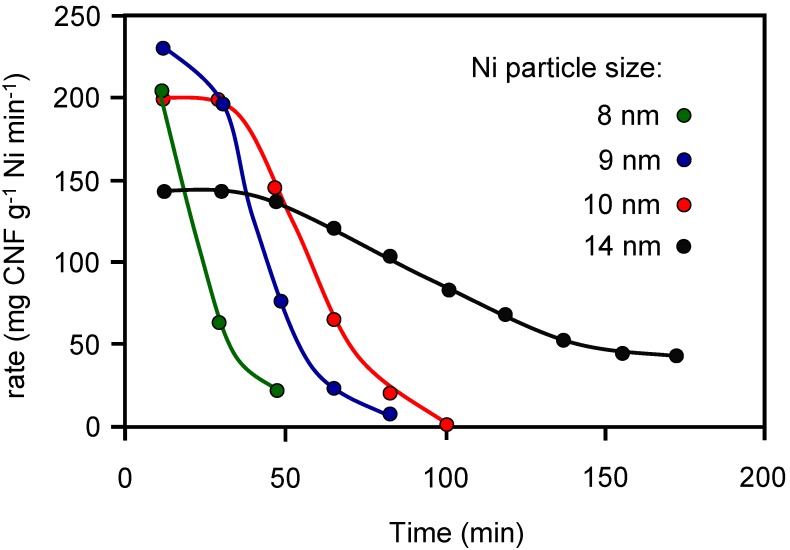
CNF growth rate at 570 ºC as a function of time on stream from a 200 ml min^-1^ gas mixture containing 50% CH_4_ and 10% H_2_ balance N_2_. Adapted from reference [26], with permission from the Royal Society of Chemistry.

The effect of the γ-Al_2_O_3_ washcoat thickness of a cordierite HM, the catalyst (Ni) content, its particle size and the type of hydrocarbon used (methane and ethene) on the final CNFs-coated HMs properties was investigated [25,26]. Bare and γ-Al_2_O_3_ wash-coated monoliths were impregnated with a Ni(II) nitrate aqueous solution to yield different Ni contents, calcined at 600 ºC and partially reduced in H_2_/N_2_ flow at 570 ºC. A thin (1 μm) CNF layer was formed on the small Ni particles covering homogeneously the channel walls provided the Ni particles were also homogeneously distributed on the washcoat. The thickness and diameter of the CNF increased when Ni particle size increased. Large Ni particles were able to grow CNFs for longer times as depicted in Figure 5, resulting in detachment of the washcoat from the cordierite, which was caused by extensive growth of CNFs out of the washcoat. In addition, extended growth of CNFs inside the cordierite body caused disintegration of the monolith body when macropores were locally overfilled with CNFs. Methane was preferred over ethene for growing CNFs because ethene grew CNFs rapidly even on relatively large Ni particles, resulting in thick fibers up to 70 nm in the macroporous cordierite, destroying the monolith. The control of Ni particle size, its distribution and the chosen hydrocarbon was essential for growing CNFs without damaging the HM.

Mullite ceramic HMs (31 and 62 cells/cm^2^) with an alumina washcoat were used to grow a CNF layer on the channel walls using a Ni catalyst and methane and propene as CNF sources [19]. Results were compared with those obtained with a cordierite HM with the same washcoat. These supports were used as bioreactors for enzyme immobilization. The high porosity of mullite compared to cordierite allowed a higher CNF deposition per unit wall volume, and so more enzyme was immobilized and more active biocatalysts were obtained with the mullite than with the cordierite HMs.

Colloidal silica was also used as washcoat for a cordierite HM (31 cells/cm^2^) [27]. After Ni deposition CNFs were grown in CH_4_/H_2_/N_2_ flow. The resulting material was also used as support for enzyme immobilization. Ni catalyst could be completely removed from the fiber tips by acid treatment, which resulted in an increase in BET surface area and pore volume. HCl and HNO_3_ treatment affected also to the fiber morphology.

CNFs were also successfully grown on a cordierite HM (62 cells/cm^2^) previously washcoated with a γ-alumina thin layer of circa 0.1 μm [28,29] that used a Ni catalyst. The entangled CNFs formed a densely packed layer with a smooth surface and with a porosity in the mesopore range. The cordierite HM must be completely covered by a thin γ-alumina layer to obtain a uniform CNF layer. In addition, the thinness of the washcoat avoid the entrapment of CNFs inside the γ-alumina pores. Using a C_2_H_6_/H_2_ gas mixture as a carbon source resulted in a more uniform CNF layer thickness and coverage when compared to a CH_4_/H_2_ mixture. The latter carbon source led to a higher carbon production rate which gave rise to less entangled CNFs. When the C_2_H_6_/H_2_ gas mixture was used both the grown conditions and the Ni catalyst particle size had a great impact on the properties of the CNF-coated HM. The growth temperature determined the CNF growth rate which influenced the mechanical strength and the CNF layer thickness of the resulting composite as shown in Table 1. The small Ni particles ensured the growth of small size CNFs.

**Table 1:** Characterization of the CNF coating on composite monoliths after growing with C_2_H_6_/H_2_ mixture at different temperatures. From reference [29], with permission from Elsevier.

Growth temperature (ºC)	CNF/monolith (wt %)	CNF/alumina (wt/wt)	Average coating thickness (μm)	Nanofiber diameter (nm)	Composite mechanical strength (MPa)
600	13.2	2.4	2.2	5–10	35
650	16	3.0	4	10–30	30
700	17.8	3.3	4	10–30	----

### 2.2. Integral carbon HMs

An alternative method to obtain carbon-based HMs is to use the carbon material or carbon precursor such as organic resins and polymers in the composition of the dough to be extruded. In this way, the carbon material or precursor is distributed homogeneously through the monolith, reducing significantly the number of steps in its preparation [2,4,31]. The dough to be extruded must have the adequate plasticity to permit their extrusion and immediate conformation in rigid structures in monolithic shape.

**Figure 6:**
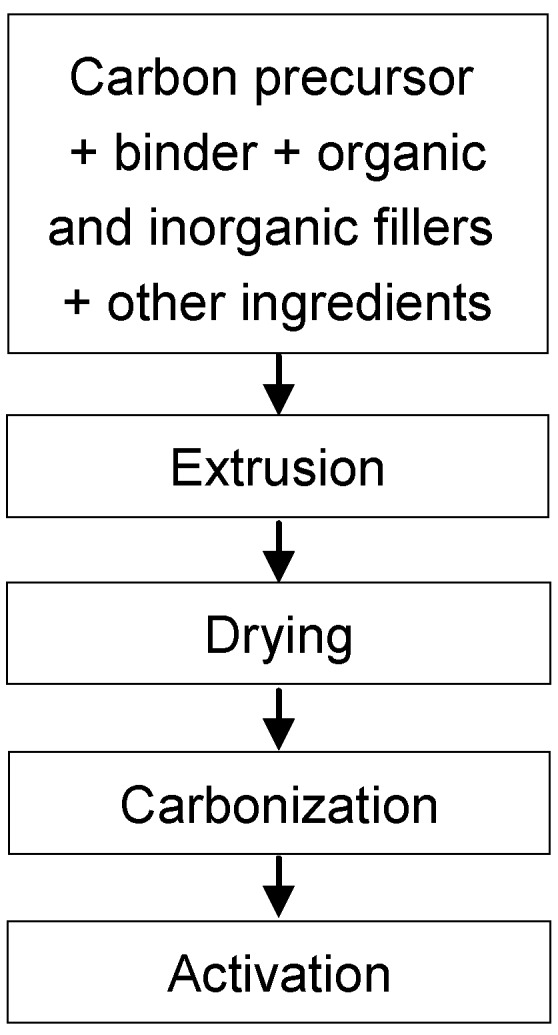
Preparation steps for integral carbon HMs

For this purpose, the carbon material or precursor is initially mixed with binders and organic or inorganic fillers. After extrusion the monoliths are dried, carbonized and sometimes activated to increase their porosity and surface area. Figure 6 shows a scheme that summarizes the preparation steps of integral carbon HMs. A preoxidation step before carbonization was reported [31] to improve the textural properties of the final carbon-based HM when coal was used as precursor. During carbonization the structure can appreciably shrink and the three-dimensional shrinkage is an important drawback. To avoid this, generally the carbonaceous material is used in combination with ceramic binders to achieve sufficient strength and stability [31,32,33].

Today, activated carbon HMs are also commercially manufactured free of binders [34,35]. These monoliths have the advantage that are electrically conducting, which makes easier the rapid volatile organic compounds (VOCs) recovery by thermal desorption at low voltage difference [36].

Fabrication of carbon honeycombs by using standard phenolic liquid resole resins as precursors was described in the literature [32]. This type of phenolic resin is very fluid, and has a viscosity of around 300–500 cP. The resin was mixed with various organic and inorganic fillers such as cordierite powder, cellulose fiber and polyester fiber to make an extrudable dough, which was then extruded through a steel die at room temperature to form the honeycomb structure. Honeycombs obtained were then dried at 90 ºC and carbonized at 900 ºC in nitrogen and activated with carbon dioxide at 700–900 ºC, depending on the composition, to obtain various burn-off levels.

Two types of samples were fabricated: type A, which involved phenolic resin as the liquid precursor, and type B, which involved the same phenolic resin but containing cobalt acetate dissolved at 1 wt % prior to mixing and fabrication of honeycomb structures. The standard honeycombs contained 50 wt % carbon on carbonization and the remaining 50 wt % of the material consisted of essentially finely ground cordierite powder. It was found that only micropores were formed in type A samples. The micropore volume increased with activation by deepening of the micropores produced during carbonization, but no micropore widening was detected even at high burn-off levels. The introduction of metal catalysts (as Co) in the resin precursor was evaluated as a means to change the pore structure of the final product. The presence of the metal catalyst changed the final pore structure significantly by the formation of a large volume of meso- and macropores, particularly at high activation levels.

**Figure 7:**
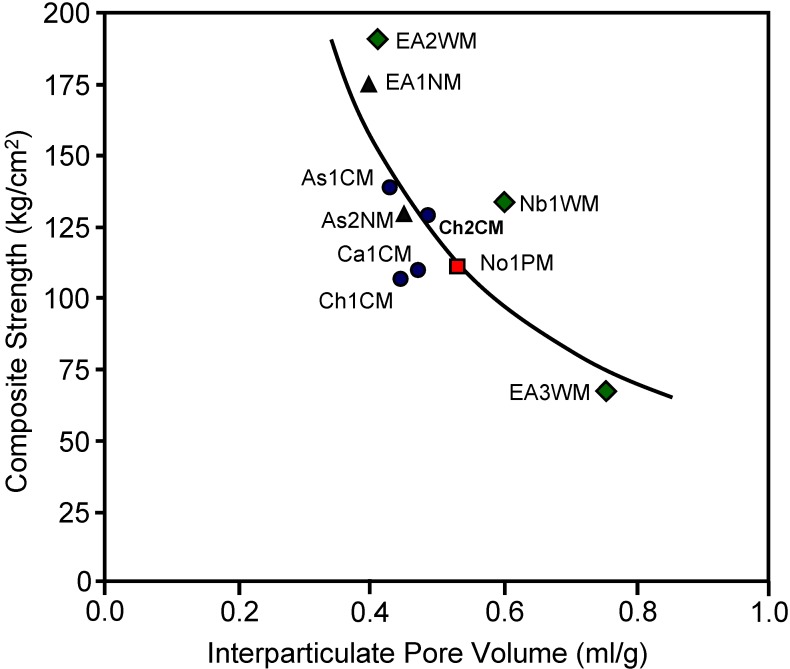
Relationship between the mechanical strength and interparticulate pore volume of the monolith composites. Origin of the commercial activated carbon used in the composite: coal (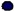), nutshell (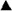), wood (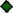) and peat (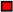). From reference [33], with permission from Elsevier.

Different commercial activated carbons to prepare integral carbon HMs were also investigated [33]. Magnesium silicate was used as an inorganic binder to improve the handling characteristics and rheological properties of the paste during the kneading and extrusion operations and to give greater mechanical strength to the final HM. An activated carbon to inorganic binder ratio of 1/1 was used. The powdered mixture was added to a kneading machine where after water addition a dough with the adequate rheological properties was formed. This dough was extruded as honeycomb monolithic structures of parallel channels of square section with a cell density of 8 cells/cm^2^ and a wall thickness of 0.9 mm. The honeycomb monoliths were dried at room temperature and then heated in air to 150 °C for four hours. Surface areas and porosities of the monoliths were reduced due to the presence of the binder in comparison with the corresponding original activated carbon. The mechanical strength of the HMs depended on the inter particle porosity (pore volume in pores greater than 17 nm) as depicted in Figure 7. These materials were also stable in air at 300 ºC.

Commercial powdered activated carbon and a natural coal were also used as carbon sources to obtain HMs [31]. A silicate clay was used as inorganic binder together with different additives to obtain doughs with adequate rheological properties. These additives are compiled in Table 2. The use of different combinations of these additives allowed improvement of the handling characteristics and rheological properties of the paste during the kneading and extrusion operations, and gave greater mechanical strength to the final heat-treated product. Monoliths with a cell density of four cells/cm^2^, in 2 × 2 and 4 × 4 configurations, and a wall thickness of 1.3 mm, were obtained. Carbonization was carried out at 840 ºC for one hour under flowing Ar. Steam activation was carried out at 860 ºC to reach a 15% burn-off. The pore texture and surface area of the resulting materials make them, according to the authors, useful as adsorbents and catalyst supports.

**Table 2:** Additives that can be used to make extrudable a carbonaceous paste. From reference [31], with permission from Elsevier.

Function	Additive
Agglomerant	Methylcellulose, starch, polyvinyl alcohol, hydroxyethyl cellulose, dextrine from potato starch
Plasticizer	Polyethilene glycol, glycerine
Defloculating	Glycerine, ammonium poliacrylate, oleic acid
Lubricant	Oleic acid, aluminium stearate, stearic acid
Dispersant	Aluminium phosphate hydrate dissolved in o-phosphoric acid, gelatine from porcine skin
Humidifying	Ethanol, kerosene
Drying	Gelatine from porcine skin, ferric chloride hexahydrate, aluminium chloride

When coals were used to prepare activated carbon HMs, both coal rank and activation time had a great impact on the pore size distribution and mechanical strength of the resulting material [37]. Thus, activated carbon HMs were prepared by extruding a mixture of a bituminous coal and organic additives followed by carbonization and steam activation [38]. A mixture of 50 wt % coal powder, 20 wt % coal tar, 2.5 wt % methylcellulose, 10 wt % bean oil and water to balance was blended and extruded into honeycomb monoliths with a wall thickness of 1.0 mm and a cell density of 8 cells/cm^2^. The extruded monoliths were dried at 120 ºC for 24 hours and then carbonized at different temperatures between 500 and 800 ºC for one hour. Monoliths were also steam activated at 850 ºC for different time periods. A high carbonization temperature resulted in chars more resistant to steam activation, and yielded carbon monoliths with less total pore volume, higher percentage of micropore volume, and higher mechanical strength. A longer steam activation time resulted in conversion of more micropores to mesopores.

Recently, integral carbon fiber HMs were obtained by vacuum moulding a mixture of carbon fibers from different origins with a phenolic resin as binder [39]. This work reports the effect of the type of carbon fiber, carbonization and activation temperature and time on the surface area and porosity of the final HMs. Table 3 shows the effect of activation time and temperature on the surface characteristics of a HM fabricated with carbon fibers from isotropic coal tar pitch.

**Table 3:** Effect of activation time and temperature on the characteristics of the carbon fiber HM. From reference [39], with permission from Elsevier.

Activation temperature (ºC)	Activation time (h)	Burn-off (%)	S_BET_ (m^2^/g)	Micropore volume (cm^3^/g)	Micropore width (nm)
850	1	8	481	0.24	2.24
850	3	19	658	0.33	1.40
850	8	47	968	0.51	1.58
950	1	31	831	0.47	1.83

## 3. Environmental Applications

Different applications of carbon-based HMs as adsorbents and support for catalysts to remove pollutants from gas streams were reported. Thus, they were used to remove VOCs from air, CO_2 _capture and NO and SO_2_ removal from flue gas, separation of CO_2_ and CH_4_ and for selective catalytic reduction (SCR) of NO with ammonia. In the next subsections, the currently reported applications of carbon-based HMs will be presented.

### 3.1. Carbon-based HMs as adsorbents

The dynamic adsorption of butane, toluene, formaldehyde, acetaldehyde and isopropanol using carbon-based HMs was studied [18]. These structures were prepared by dip-coating ceramic HMs with a phenolic resole of low viscosity (100 cP) that were carbonized at 900 ºC in N_2_. High adsorption efficiencies were obtained even with high flow rate and low concentration streams. Adsorption efficiencies and capacities could be controlled by adjusting wall thickness. In addition, it was shown that space velocity should be used instead of surface velocity to characterize adsorption performance.

A comparative study on the adsorption of n-butane from air on an activated carbon monolith and in granular form was carried out by Crittenden *et al.* [34]. The carbon square channel monolith from MAST Carbon had a cell density of around 89 cells/cm^2^, with a cell size of 0.63 mm and a wall thickness of 0.43 mm. Granules with sizes between 600 and 1400 μm were obtained by crushing samples of the monolith. Results were obtained using a dynamic flow apparatus and showed that it was possible to manufacture an activated carbon monolith that had a capacity and dynamic mass transfer performance equal to that of the equivalent mass of granules. The pressure drop, however, was less than 6% of that of the granule beds.

The effect of cell density, stacking and total length of carbon-coated HMs on the breakthrough curves of n-butane diluted (7 g/m^3^) in an air flow of 1500 cm^3^/min was reported [40]. The carbon-coated HM was prepared by dip-coating the ceramic monolith in a phenol novolac resin. Carbonization was carried out at 700 ºC and the resulting material was steam activated at the same temperature. Table 4 shows the width values of the breakthrough profile (time difference for outlet concentrations of C/C_0_ = 0.95 and C/C_0_ = 0.05). For each cell density value, the increase in total length produced an increase in the breakthrough profile width. In addition, for each total length the increase in cell density produced a decrease in the breakthrough profile width.

**Table 4:** Breakthrough profile width t_0.95_-t_0.05_ (min). From reference [40], with permission from Elsevier.

Cell density – individual piece length	Total length (cm)		
	5	10	15
31 cells/cm^2^ – 5 cm	31.7	41.7	46.7
62 cells/cm^2^ – 5 cm	24.3	36.2	45.0
93 cells/cm^2^ – 2.5 cm	15.3	22.4	31.4
140 cells/cm^2^ – 1 cm	16.5	20.2	31.9

**Figure 8:**
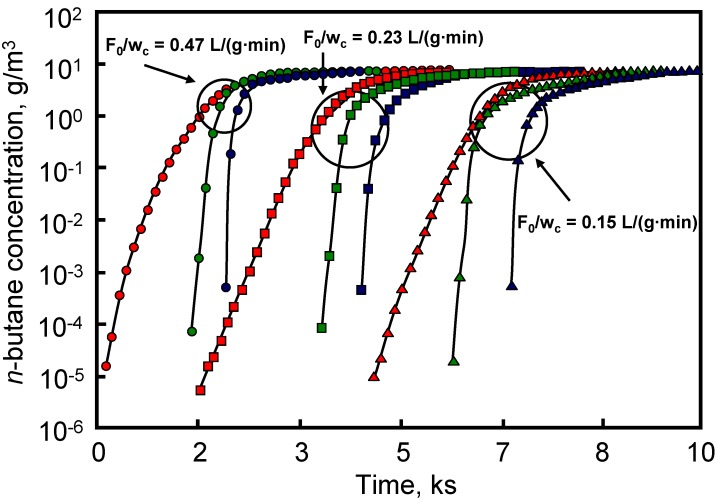
Breakthrough profiles for carbon-packed beds and monoliths (31 and 62 cells/cm^2^) (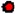, Norit R1 4.3 g; 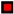, Norit R1 8.6 g; 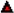, Norit R1 13 g; 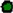, (31 cells/cm^2^)-5 cm-5 cm; 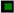, (31 cells/cm^2^)-5 cm-10 cm; 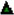, (31 cells/cm^2^)-5 cm-15 cm; 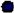, (62 cells/cm^2^)-5 cm-5 cm; 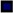, (62 cells/cm^2^)-5 cm-10 cm; 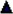, (62 cells/cm^2^)-5 cm-15 cm. From reference [40], with permission from Elsevier.

The performance of activated carbon-packed beds was compared with that of the carbon-coated HMs and results are depicted in Figure 8. This Figure shows that carbon-coated HMs had a sharper breakthrough profile, with the breakthrough point occurring later than on the activated carbon-packed beds. This behavior was associated with the short diffusion length in the monoliths. This is interesting with respect to the application of the monoliths in gas mask canisters, because breakthrough time is delayed with regard to that found for activated carbon-packed beds.

**Figure 9:**
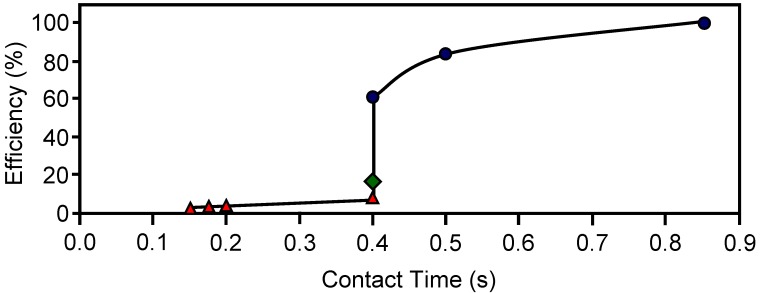
The dynamic adsorption efficiency towards o-DCB for different contact times and temperatures: 30 ºC (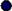), 100 ºC (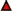) and 150 ºC (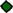). From reference [43], with permission from Elsevier.

Carbon-based HMs prepared from different activated carbons mixed with a silicate (binder) were used to remove o-dichlorobenzene from air streams at different temperatures and linear velocities. The adsorption capacity of these HMs was also studied under static conditions [41,42,43]. The carbon-based HMs had square section channels with cell density of 8 cells/cm^2^ and a wall thickness of 0.9 mm. Adsorption capacity at 30 ºC could be related with the micropore volume of the carbon-based HMs. The dynamic adsorption efficiency is depicted in Figure 9. When adsorption was carried out at 30 ºC the efficiency decreased from 100 to 60% while contact time diminished from 0.9 to 0.4 s. Therefore, the efficiency of these HMs could be improved by reducing the open channel width. Figure 9 also shows a severe decrease in efficiency when adsorption temperature increased from 30 to 150 ºC.

The static adsorption of different organic compounds and water vapor on various HMs prepared from commercially available activated carbons, alumina and titania using a silicate clay as binder was also reported [44]. When the pretreatment temperature of the carbon-based HM increased, the organic vapor adsorption rate also increased, whereas the water adsorption rate decreased. However, for the alumina and titania HMs, the increase in pretreatment temperature did not affect the organic vapor adsorption rate, but decreased the water adsorption rate. These results were explained as to the result of changes in surface hydrophobicity with pretreatment temperature.

The dynamic adsorption/desorption of o-xylene on an integral carbon honeycomb monolith, which was prepared from a bituminous coal and using some additives to obtain the adequate rheological properties for extrusion, was studied [45]. The o-xylene adsorption was carried out at 27 ºC from a N_2_ flow (100 cm^3^/min) containing 0.36% of the pollutant. Total adsorption capacity was around 550 μmol/g, similar to that reported for powder-activated carbons. The adsorption capacity remained constant after several adsorption/desorption cycles.

An integral carbon honeycomb monolith from MAST Carbon was used to adsorb CO_2_, CH_4_ and N_2_ [46]. Adsorption equilibrium was measured over a pressure range between 0 and 700 kPa and at temperatures between 26 and 149 ºC. Results obtained were well fitted by the multisite Langmuir model. The selectivity toward carbon dioxide decreased when the pressure increased. The diffusion of simple gases in the porous structure of the activated carbon honeycomb monolith was studied by diluted breakthrough experiments. The diffusivity coefficients obtained resulted from a combination of macro and micropore diffusivities and they had and exponential dependence with temperature.

**Figure 10:**
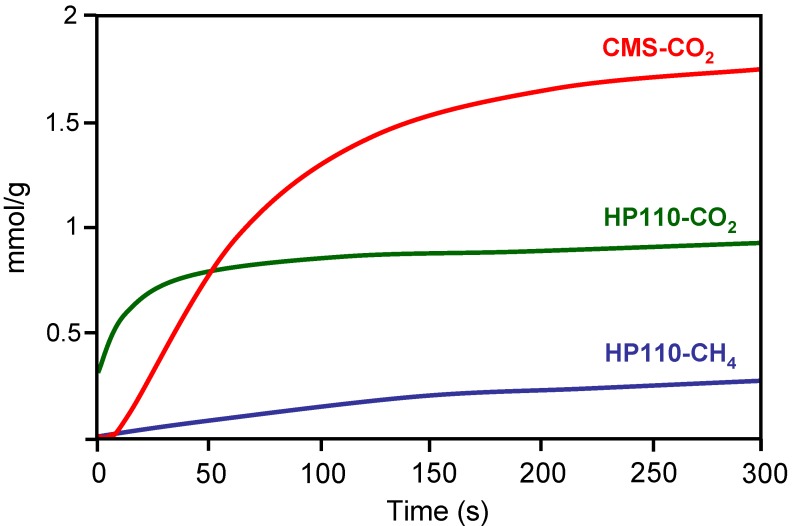
Kinetics of CO_2_ and CH_4_ adsorption on carbon honeycomb HP110 and a commercial carbon molecular sieve CMS. From reference [24], with permission from Elsevier.

The molecular sieve properties of a carbon-based HM was compared with that of a commercial carbon molecular sieve (CMS) [24]. For this purpose, the CH_4_ and CO_2_ adsorption kinetics were studied. The carbon-based HM was prepared by impregnation with a petroleum pitch of a cellulose-based corrugated paper. After stabilization in air (300 ºC), the resulting material was carbonized at 1000 ºC in N_2 _flow and CO_2_ activated at 870 ºC to different burn-off degrees. CH_4_ and CO_2_ adsorption kinetics was carried out at constant volume in a high-pressure thermobalance at 0.1 MPa and 25 ºC. Results obtained with one of the carbon-based HM and the CMS are depicted in Figure 10. The CMS showed good molecular sieving properties since they did not adsorb CH_4_ but showed a large CO_2_ adsorption capacity. However, the carbon-based HM showed faster initial CO_2_ adsorption than CMS. According to the authors this is of great significance for the application in pressure swing adsorption (PSA), where it is more appropriate to reach the adsorption stage in a shorter time from an economical point of view.

An electric swing adsorption (ESA) technology, which used an integral activated carbon HM from MAST Carbon as adsorbent, was employed to remove low molar fractions of CO_2_ in flue gas streams [47]. The HM (3 cm diameter, 30 cells/cm^2^ and 0.11 mm wall thickness) had low electrical resistivity that enabled an enhanced CO_2_ desorption rate by heating the HM passing an electrical current through it. Authors proposed a mathematical model for the HM used and different ESA cycles for CO_2_ captures.

The ESA technology was also applied to remove toluene from a N_2_ flow by using an integral activated carbon HM with 16 square cells/cm^2^[48]. The HM electrical resistivity decreased as temperature and uptake increased. The concentration of the desorbed toluene as function of time had the shape of a peak followed by a tail, which was the main drawback of ESA. Authors indicate that this technology is better to use it for treatments at moderate concentrations.

The use of integral carbon fiber HMs for CO_2_ capture from flue gas produced by power plants was recently reported [39]. This work shows the optimal fabrication parameters for the carbon fiber HM based on maximal CO_2_ adsorption performance, which reached a value of 103.5 cm^3^ STP/g at 0 ºC. Adsorption breakthrough tests showed that the HM could reduce the CO_2_ inlet concentration from 9.7% to 0.29% in the outlet.

An integral carbon HM with 4.6 cells/cm^2^ and wall thickness of 1.6 mm was prepared from a mixture of a sub-bituminous coal char and carboxymethylcellulose [49]. This HM was used to study the dynamic adsorption of NO from Ar flow. The NO adsorption capacity of the carbon-based HM was greater than that of powder activated carbons. In addition, the presence of Cu in the HM or O_2_ in the gas stream favored the NO adsorption.

### 3.2. Carbon-based HMs as supports for catalysts

An efficient technology to reduce nitrogen oxide emissions from stationary sources is the SCR with ammonia. The industrial operations are generally carried out using a V_2_O_5_/TiO_2_ catalyst in monolithic form at temperatures between 300 and 500 ºC [50]. However, in this temperature range SO_2_ and particle poisoning are very serious drawbacks. So, there is interest to develop low-temperature catalysts that could be used for simultaneous NO and SO_2_ removal from flue gas streams at temperatures below 250 ºC, which would be more economic than individual separation of both contaminants. The use of carbon-based HMs as supports for vanadium catalysts was also reported in the literature [51,52,53,54,55,56,57,58,59,60,61,62]. These studies were carried out with both dip-coated and integral carbon HMs and they dealt with the effect of the carbon coating pore texture and thickness, oxidation pretreatments, vanadium content, promotion of a second metal, previous sulfation of the vanadium catalyst and presence of SO_2_ and H_2_O in the gas feed on the catalyst dispersion and activity and selectivity in the SCR of NO.

When vanadium supported on carbon-coated HMs (62 cell/cm^2^) were used it was found that a high mesopore volume of the carbon coating increased the surface oxygen complexes fixed after an oxidation treatment. This was essential to obtain a high vanadium dispersion and a high activity in the SCR of NO. Conversely, the micropores did not contribute to enhance the vanadium dispersion [51].

Then a mesoporous carbon-coated HM was used to deposit different vanadium loadings [52,53] that was dispersed as V_2_O_5_ overlays up to 6 wt % loading. For higher loadings vanadia crystallites appeared. Both activity and selectivity to N_2_ in the SCR of NO dropped dramatically for loadings surpassing the monolayer coverage. The turnover frequency of the catalysts was lower than that of TiO_2_-supported catalysts. However, the carbon-supported vanadia catalysts were not deactivated by SO_2_ at low reaction temperatures, while the corresponding titania-supported catalysts were readily deactivated. Finally, an optimal carbon-coating thickness of around 30–40 μm was found by simulating, with a one-dimensional catalyst model, the effects of thickness on the geometric parameters and conversion.

The effect of oxidation of carbon-coated HMs on vanadium dispersion and catalytic activity in the SCR of NO was also studied [54,55,56]. Preparation of vanadia catalysts was carried out by equilibrium adsorption impregnation with VO^2+^ of a dip-coated HM (31 cells/cm^2^) that was previously oxidized with HNO_3_ and ionic exchanged with a NaOH solution [54]. This preparation method produced an increase in the catalytic activity due to an increase in vanadium content and dispersion. 

In other works [55,56] oxidation of the dip-coated HM (62 cell/cm^2^) was carried out with different oxidizing agents, and vanadium catalysts were prepared by ion-exchange with VO^2+^ ions. Treatments led to high vanadium dispersion except in the case of ozone treatment. For well dispersed catalysts, their activity linearly increased when vanadium loading increased. Selectivity to N_2_ was 100% in all catalysts. The acidic character of the surface was of great importance for achieving a high NO reduction efficiency. Thus, a high surface acidity led to a strong NH_3_ adsorption on the carbon surface decreasing the NO conversion. Conversely, a low surface acidity did not promote the vanadium fixation and dispersion, resulting in a decrease of NO reduction efficiency.

The addition of a second metal (Fe, Cr, Cu or Mn) as promoter to vanadium catalysts supported on mesoporous carbon-coated HMs resulted in a modest increase in their specific activity [57]. However the introduction of 400 ppm of SO_2_ in the gas feed increased three times the activity of the catalysts, which did not change in 20 hours operation at 150 and 180 ºC (see Table 5). This was attributed by the authors to the accumulation of NH_4_HSO_4_ in the micropores but not in the mesopores (predominant in the carbon coating) where most of vanadium was located.

**Table 5:** Turnover frequency at steady state in the SCR of NO with NH_3_ before and after addition of 400 ppm SO_2_. From reference [57], with permission from Elsevier.

Reaction temperature (ºC)	TOF (s^-1^) x 10^–4^		
	With fresh catalyst	20h Time-on-stream with SO_2_	
150	1.7	4.9	
180	4.9	9.6	

A vanadia catalyst supported on a carbon-coated HM (31 cells/cm^2^) was used to treat the exit gases of a coal-fired power plant [54]. The catalyst was placed in the outlet pipe where temperature was about 150 ºC. Some values of the operation parameters of the power plant were: gas flow 1.3 × 10^6^ m^3^/h, 200–700 ppm NO, 100–350 ppm SO_2_ and 6–10% O_2_. After 24hours, the conversion dropped to 40% of the initial value, and after 200hours, conversion remained constant at 13% of the initial value. Loss of activity was due to poisoning by As and sulfate formation.

Wang *et al*. [58,59] studied, at laboratory scale, the simultaneous NO and SO_2_ removal at low temperatures using a vanadia catalyst supported on an integral activated carbon HM (16 cells/cm^2^). Catalysts with 1–2 wt % vanadia and at 200 ºC showed high activity in the simultaneous NO and SO_2_ removal. Regeneration of the catalysts produced a high increase in the SCR of NO with NH_3_ and a low increase in the SO_2_ removal. Some components of the binder used such as silica, alumina, alkaline and alkaline-earth oxides were unfavorable for NO and SO_2_ removal.

Sulfation of vanadia supported carbon-coated HM resulted (62 cell/cm^2^), after an induction period, in a much higher conversion than the fresh catalyst in the SCR of NO at low temperatures (<200 ºC) [60]. Sulfates were anchored to carbon in the vicinity of vanadyl sites. This improved the redox properties in the sulfated catalyst, which caused its superior performance compared to the fresh one. When either water or SO_2_ was added to the gas feed the sulfated catalyst kept a 100% conversion to N_2_ in the low temperature range (200–230 ºC). Conversely, when both water and SO_2_ were added simultaneously the conversion and selectivity decreased, because water sped the ammonium sulfate deposition. This sulfated catalyst had higher activity at low temperature than commercial VO_x_/TiO_2_ catalysts and, in addition, the presence of water in the gas feed did not inhibit its activity [61].

The influence of the coating (carbon or alumina) of a cordierite HM on the behavior of vanadia catalysts for the SCR of NO with ammonia was recently reported [62]. Results found showed that both kinds of catalysts had similar activity under steady-state conditions. However, the carbon coating was much thinner, better adhered to the cordierite, provided a higher surface area, and had higher thermal shock and vibration resistance than the alumina coating. 

Mn and Cu oxides supported on carbon-based HMs have been also used as catalysts for the SCR of NO with NH_3_[63,64,65]. A carbon-coated HM (31 cells/cm^2^) was used as support of manganese oxide [63], which showed good activity, 60–70% NO reduction at 150 °C. Support oxidation enhanced the Mn loading and activity. A high active catalyst (more than 90% conversion at 150–200 ºC) was also obtained with other Mn oxide supported on a dip-coated HM [64]. The SCR of NO was improved by doping with Ce and Pd. In addition, the tolerance to SO_2_ increased after doping with Fe and V.

On the other hand, Mn and Cu were used as catalysts supported on integral carbon HMs (13.7 cells/cm^2^ and 0.8 mm wall thickness) [65]. The most relevant results, according to the authors, were that microwave drying of the impregnated monoliths led to better metal dispersion and distribution and also to differences in the metallic phases formed compared to conventional drying methods. Some of the Cu catalysts were very active at low temperatures and stable upon consecutive cycles and with time.

Catalytic combustion is one of the most important technologies for eliminating VOCs present at low concentration in effluent streams. From an energetic point of view, and to avoid NO_x_ formation, low temperatures (below 200 ºC) are preferred. However, at these conditions, water vapor produced during combustion can be retained on the catalytic support, with negative effects on the activity of the catalyst. Hydrophobic catalyst supports may overcome this effect, so the tunable surface hydrophobicity of carbon materials has been an important consideration for their application as catalyst support in VOCs combustion.

The catalytic combustion of benzene, toluene and xylenes (BTX) using Pd and Pt supported on different carbon-based HMs was reported [30,66,67,68]. Some supports were prepared from a commercial cordierite HM of 5 cm length, 1 cm diameter, 62 cells/cm^2^ and 0.18 mm wall thickness. A carbon layer was deposited on the channel walls, by the dip-coating method, of either the cordierite support or the cordierite support previously modified with alumina to block its macroporosity. Another carbon-based HM was prepared by CNF growing on the above alumina coated cordierite HM. Finally, two commercial integral HMs were also used with the same geometric characteristics than the above cordierite HM. Pd and Pt catalysts were prepared by impregnation with an aqueous solution of the corresponding tetraammine metal (II) nitrates.

**Table 6:** Surface characteristics of the carbon-based HMs. Values in parenthesis are given per gram of carbon. From reference [67], with permission from Elsevier.

Support	S_BET_ (m^2^/g)	S_External_ (m^2^/g)	V_Macro_(cm^3^/g)	V_Meso_ (cm^3^/g)
WA	474 (1366)	4 (12)	0.325 (0.937)	0
WB	460 (1489)	62 (199)	0.233 (0.754)	0.138 (0.447)
HPM	(2)	(<1)	non detected	non detected

**Figure 11:**
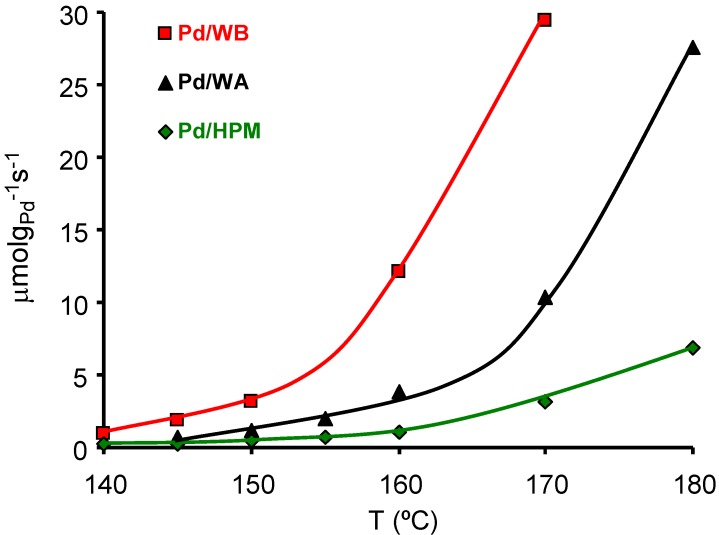
Activity of the catalysts in the m-xylene combustion (μmol of m-xylene burned per gram of Pd and per second). From reference [67], with permission of Elsevier.

The monolithic catalysts were stable during the reaction and no gasification of the carbon-coated monoliths was observed during the catalytic combustion of xylenes in the temperature range studied (120–180 ºC). Pt catalysts were more active than the Pd ones. Pt catalysts with higher metal particle size were more active whereas the opposite was observed with Pd catalysts. Complete xylene combustion was reached in the temperature range studied with total conversion to CO_2_ and H_2_O [66]. The effect of carbon coating porosity on the Pd catalyzed m-xylene combustion was studied [67] with different carbon-coated HMs whose surface area and porosity are compiled in Table 6. Samples WA and WB were two integral carbon HMs with a total carbon content of 34.7 and 30.9 wt %, from MeadWestvaco Corporation. Sample HPM was a carbon-coated HM with a total carbon content of 6.3 wt %. The activity of the Pd catalysts with close Pd content (around 0.4 wt %) supported on the above supports is depicted in Figure 11. These results show that the carbon external surface area, the macro and mainly mesopores, play an important role in this reaction, improving the contact between the Pd particles and the *m*-xylene molecules. Thus, for similar Pd loading and Pd particle size the larger the macro- and mesoporous surface area the higher the activity.

The catalytic behavior of Pd and Pt supported on CNF-coated HMs was studied in the low-temperature catalytic combustion of BTX, and compared with the performance of the above metals supported on alumina-coated HMs [30]. The catalyst supported on the CNF-coated HMs was the most active, independent of the metal catalyst or the tested aromatic compound as indicated by the TOF values obtained at low conversion that are shown in Table 7. This was due to the fact that the CNF surface was more hydrophobic than that of alumina, and the release of water molecules produced during the combustion was favored. One of Pt catalysts supported showed the best performance, and was already active at 120 ºC in the benzene combustion.

**Table 7:** TOF [×10^3^] data (s^-1^) for the catalysts in the benzene combustion at 150 ºC, and for toluene and m-xylene at 160 ºC. From reference [30], with permission from Elsevier.

Catalyst	Benzene	Toluene	m-Xylene
Pt/CNF	5.07	2.79	1.95
Pd/CNF	1.38	0.62	0.56
Pt/Alumina	0.24	0.55	0.46
Pd/Alumina	0.16	0.40	0.23

BTX combustion reactions were catalyzed by Pt and Pd through different kinetic mechanisms, which explained according to the authors why Pt catalysts were always more active than the Pd ones deposited on the same support [30,68].

## 4. Conclusions

Carbon-based HMs are very attractive for environmental gas-phase applications when large gas flows have to be treated, due to the good properties of carbon as an adsorbent and support for catalysts. These materials can be either carbon-coated or integral carbon HMs. The first ones are prepared by depositing a thin carbon layer on the channel walls of a cordierite HM by dip-coating or CVD.

To obtain a homogeneous carbon layer covering the channel walls by dip-coating, the election of the carbon precursor, its proportion and viscosity, the removal of the excess solution after the dip-coating step and the dip-coating temperature must be controlled. CVD gives place to CNF growth on the channel walls from hydrocarbon/H_2_ mixtures. In all cases the ceramic HMs are previously washcoated with alumina or silica to prevent the CNF growth inside the macropores of the HM destroying the monolith. The structure and dimensions of the CNFs are determined by the temperature, gas composition, metal catalyst, metal particle size and nature of the support and washcoat. The support structure is determined by the extent of the fiber entanglement and the individual fiber orientation.

Integral carbon-based HMs are prepared using carbon precursors such as organic resins, polymers, activated carbons and coal in the appropriate composition for the dough to be extruded. In this way the carbon material is distributed homogeneously through the monolith, reducing significantly the number of steps in its preparation. The dough to be extruded must have the adequate plasticity to permit their extrusion and immediate conformation in rigid structures in monolithic shape.

So far, the main applications as adsorbent were the removal of different VOCs from air, CO_2_ capture and NO and SO_2_ removal from flue gas and the separation of CO_2_ and CH_4_. Carbon-based HMs had sharper breakthrough profiles than activated carbon packed beds. Catalytic applications were VOCs combustion and the selective catalytic reduction of NO with ammonia in the absence and in the presence of SO_2_, where vanadia supported on carbon-based HMs have shown to be very active catalysts at low temperatures and resistant to SO_2_ poisoning. Surface area and porosity of carbon-based HMs controlled the adsorptive properties and dispersion of the catalysts. In addition, hydrophobicity of carbon-based HMs was a very important parameter of these structures that influenced water adsorption rate compared to other HMs prepared with alumina or titania. Carbon hydrophobicity was also very convenient in VOCs combustion to avoid the deactivation of the supported catalysts. 

Finally, a challenge to be addressed with carbon-based HMs is their use in environmental aqueous phase reactions, because to our best knowledge there are no references in the literature on this application, although other catalytic reactions have been carried out in that phase. We think that these HMs should be investigated in this application due to carbon materials are well known as adsorbents in liquid phase. In addition, they also play an important role as catalysts in advanced oxidation processes, which involve degradation and oxidation of micropollutants by hydroxyl radicals. 
